# Past, Present and Future of Colorectal Cancer in the Kingdom of Saudi Arabia

**DOI:** 10.4103/1319-3767.43275

**Published:** 2008-10

**Authors:** Ezzeldin M. Ibrahim, Ahmed A. Zeeneldin, Tawfik R. El-Khodary, Aboelkhair M. Al-Gahmi, Bakr M. Bin Sadiq

**Affiliations:** Department of Oncology and Hematology, King Faisal Specialist Hospital and Research Centre, Jeddah, Kingdom of Saudi Arabia; 1 Research Centre, King Faisal Specialist Hospital and Research Centre, Jeddah, Kingdom of Saudi Arabia

**Keywords:** Colorectal cancer, developing country, incidence, Saudi Arabia

## Abstract

**Background/Aims:**

The crude frequency of colorectal cancer (CRC) is second to breast cancer in the Kingdom of Saudi Arabia (KSA). To assess the future burden of CRC in the country, we designed a model that takes into consideration the recent lifestyle pattern and the growth and aging of the population.

**Methods:**

We compared CRC statistics for KSA (using data from the National Cancer Registry) with that from the Surveillance, Epidemiology and End Results (SEER) databases of the United States of America (USA). We used the Joinpoint regression program to identify changes in secular trends, while the GLOBOCAN 2002 software was used to project future incidence and mortality.

**Results:**

Between 1994 and 2003, age-standardized rates (ASRs) for CRC in KSA almost doubled, as compared to a nonsignificant decline in USA. Between 2001 and 2003, while the annual percent change (APC) of CRC incidence in the USA showed a nonsignificant decrease in females, APC in Saudi females showed a nonsignificant rise of six percent. On the other hand, the rising incidence among Saudi males, during the years 1999 to 2003, was significant, with an APC of 20.5%. The projection model suggested that the incidence of CRC in KSA could increase fourfold in both genders by the year 2030.

**Conclusions:**

In KSA, the present and expected increase in CRC rates is alarming. Pragmatic recommendations to face that challenge are discussed. The present work could serve as a model to study other prevalent types of cancer, particularly in developing countries.

In the year 2002, colorectal cancer (CRC) was the third and fourth most common cancer in females and males, respectively, worldwide.[1] Its prevalence is second only to that of breast cancer, with an estimated 2.8 million persons alive with CRC within five years of diagnosis. The highest incidence rates occurred in North America, Australia, Western Europe and Japan. The incidence tends to be low in Africa and Asia and intermediate in the southern parts of South America. Although the Kingdom of Saudi Arabia (KSA) is considered a low-incidence area for CRC, the disease ranks second, after breast cancer, constituting almost nine percent of the newly diagnosed cases, ranking first and third among the male and female population, respectively.[2]

Geographic differences for CRC are probably explained by dietary and other environmental exposures.[1] This is supported by studies of migrants moving from low-risk to high-risk areas.[3,4] A higher risk of CRC was found in subjects consuming a diet poor in fiber[5] and rich in meat[6] and fat.[7] Physical inactivity, excess body weight, and a central deposition of adiposity have a major influence on the risk of CRC.[8–11] Non-dietary causes of CRC include genetic predisposition,[12] Crohn's disease and ulcerative colitis.[13]

Changing trends in the incidence and mortality of CRC have been shown in many high- and low-rate areas. The incidence rates of CRC are increasing rather rapidly in countries where the overall risk was formerly low.[13] For mortality, the pattern is similar, with an increase in the countries with a low initial rate, small increases or stable rates in countries with moderate rates, and a decrease for high-rate populations.[13–15]

The reasons for these changes are certainly multiple.[13] However, the principal cause of the increased risk in the countries of Eastern Europe and Asia is probably westernization of their way of life, particularly with respect to diet.[16] The converse effect, with some improvements in the quality of the diet in younger generations, may explain the observation, notably in the USA, of cohort effects with a decrease in the incidence rates among the younger age groups.[17–19] The changes in mortality may be consequent to changes in incidence, improvements in the treatment, or, as in the USA, improved early detection, probably due to screening examinations and detection of premalignant lesions.[20,21]

Comparisons of cancer rates, including CRC, across time or region, are complicated by variation in the age structures of the different populations. Age-standardized rate (ASR), a summary measure of the cancer rate that a population would have if it had a standard age-structure, is employed to compensate for such variations. The world standard population (WSP) is the most frequently used standard for international comparisons.[22]

The purpose of this work is to analyze the rates of CRC in KSA, to examine its time trends, and to compare the disease pattern with that in the USA. With a view to providing pragmatic recommendations to the healthcare community, the future burden of CRC in KSA is projected.

## PATIENTS AND METHODS

Age-standardized rates (ASRs) for CRC in KSA, during the calendar period 1994 to 2003, were calculated from the National Cancer Registry (NCR) database adjusted for WSP 2000. National Cancer Registry is a population-based registry; it commenced reporting cancer in January 1994.[2] For USA, ASRs per 100,000 population-year were calculated with SEER*Stat 6.2.3.,[23] using information from the Surveillance, Epidemiology, and End Results (SEER) databases adjusted for the WSP 2000.

SEER's Joinpoint regression program was used to identify changes in secular trends and to determine whether apparent changes in the trend data are statistically significant and two-sided, and P < 0.05.[24] The software takes the annual rate data and fits a series of joined straight lines on a log scale to the trends in the rates. The tests of significance use a Monte Carlo Permutation method. In this report, the resultant trends of varying times were described by the annual percent change (APC).

To predict CRC incidence in KSA, we used the GLOBOCAN 2002 database software that estimated incidence and prevalence of and mortality from 27 cancers for all countries in the world in 2002.[25] The GLOBCAN database was also used to compare incidence and mortality statistics for CRC in the country against that in various geographical regions. GLOBCAN 2002 data for CRC in KSA was slightly higher than that reported by the NCR, to account for potential under- and/or delayed reporting in the local registry.

## RESULTS

### Incidence data

**Figure 1:**
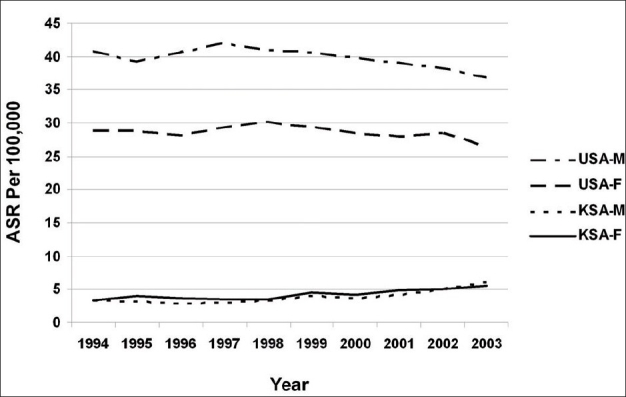
The age-standardized rate for colorectal cancer for males and females in the Kingdom of Saudi Arabia and the USA (1994-2003). M and F denote males and females, respectively

Table 1 depicts a significant difference in the incidence rates for CRC in the USA from 1994 to 2003, as against that in KSA during the same period. Also shown is the higher incidence among males, as against females, in USA, a pattern that is not apparent in KSA [Fig. 1]. Table 1 also shows that there is a recent rise in incidence rates for both genders in KSA.

**Table 1:** ASR for colorectal cancer (1994-2003) in the Kingdom of Saudi Arabia and the United States of America

	ASR for Incidence (KSA)			ASR for Incidence (USA)		
		
Year	Males	Females	ALL	Males	Females	ALL
1994	3.36	3.45	3.38	40.80	28.90	33.41
1995	3.25	4.02	3.56	39.20	28.80	32.63
1996	2.93	3.73	3.25	40.70	28.20	33.01
1997	3.05	3.51	3.22	41.90	29.30	34.06
1998	3.45	3.52	3.48	41.00	30.20	34.14
1999	3.98	4.63	4.26	40.60	29.40	33.59
2000	3.70	4.28	3.95	39.70	28.50	32.82
2001	4.15	4.92	4.48	39.10	28.00	32.24
2002	5.09	5.07	5.07	38.20	28.40	32.11
2003	6.06	5.60	5.84	36.90	26.50	30.49

ASR- age-standardized rate, KSA- Kingdom of Saudi Arabia, USA- United States of america

Table 2 shows the summary statistics for the ASR fitting of trends using the Joinpoint software for USA and KSA patients. In USA, while the APCs in recent years (2001 to 2003) showed a 3 and 2.5% decrease for males and females respectively, the changes were not significant. In KSA, the trend for females demonstrates a rise since 1998; however, the rise was not significant. On the other hand, the rising incidence among Saudi males from 1999 to 2003 was significant, with an APC of 20.5%.

**Table 2:** Trends in age-standardized incidence rates for colorectal cancer in the United States of America and the Kingdom of Saudi Arabia, in 1994-2003

	United States of America				Kingdom of Saudi Arabia			
		
Line segment	Year	APC (Males)	Year	APC (Females)	Year	APC (Males)	Year	APC (Females)
Segment 1	94-97	1.31	94-96	-0.44	94-96	-6.6	94-98	0.42
Segment 1	97-01	-1.38	96-99	1.24	96-99	7.2	98-01	9.68
Segment 3	01-03	-2.95	99-03	-2.51	99-03	20.5†	01-03	6.06

APC- Annual percent change

† Significant APC

### KSA CRC ASR: Statistics pertaining to incidence and mortality and comparison with various geographical regions

GLOBCAN 2002 data for CRC in KSA is slightly higher than that reported by the NCR, to account for potential under- and/or delayed reporting in the local registry. Using GLOBCAN, we compared the ASR for the incidence of CRC and mortality for KSA in the year 2002, as seen against different geographical regions [Table 3]. The incidence of CRC in males and females in KSA is almost half and one-third of that for the whole world and the more developed countries, respectively. On the other hand, while the incidence of CRC in Saudi males was equal to that in the less developed countries, the incidence in females was higher. Similarly, mortality statistics for Saudi males and females was less than that for the whole world and the more developed countries. Nevertheless, while CRC mortality for Saudi males was equivalent to that in the less developed countries, the mortality in females was higher.

**Table 3:** Comparison of colorectal cancer (incidence and mortality) in the Kingdom of Saudi Arabia in the year 2002, with various geographical regions

	KSA Males %	KSA Females %
CRC* Incidence		
KSA† vs World	51	62
KSA vs Less developed countries	94	112
KSA vs More developed countries	26	34
CRC mortality		
KSA vs World	65	77
KSA vs Less developed countries	99	120
KSA vs More developed countries	38	49

* CRC- Colorectal cancer

† KSA- Kingdom of Saudi Arabia

### Future CRC burden in KSA

According to the USA Census Bureau International Database, by the year 2010, 2015 and 2020, the total male population of KSA is expected to be 15, 17, and 19 million respectively, while the expected female population would be 13, 15, and 17 million respectively.[26] The median age of the Saudi population is projected to increase from the current 21 years to 23, 26, and 28 years in 2010, 2015, and 2020, respectively.

Predicting the future CRC burden in KSA was projected using conservative estimates of an APC increase of 0 to 2% in ASR. The projected increase in APC was modeled considering recent trends and future changes in the demographic parameters. Table 4 demonstrates the expected increase in the incidence of CRC in the future decades. It appears from Table 4 that the incidence could increase in both genders by almost fourfold by the year 2030.

**Table 4:** Predicted colorectal cancer burden in the Kingdom of Saudi Arabia, up to 2030

Year	Males		Females	
		
	No. of patients	% Changes from 2005	No. of patients	% Changes from 2005
2005	680	-	537	-
2010	881	30	697	30
2015	1,192	80	931	70
2020	1,792	170	1,397	160
2030	3,171	370	2,538	370

## DISCUSSION

In 2003, CRC was the first and third among male and female population, respectively, accounting for 9% of all newly diagnosed cases with overall, female and male ASRs of 6.6, 5.9 and 7.3 per 100 000 population, in the Kingdom of Saudi Arabia.[2]

In the current study, we demonstrated that ASRs of CRC in KSA between 1994 and 2003 was progressively rising (almost doubling) both in males and females. This is in contrast to what was happening in the USA, where the rates were declining. The decline in the incidence of CRC in USA is likely to be associated with disease prevention through screening[27,28] and removal of precancerous polyps.[29,30] The fact also remains that improvement in the quality of the diet, through decreasing the consumption of animal fat and red, and increasing the consumption of vegetable and fruit, is a significant factor.[31] In KSA, progressively increasing exposure to risk factors, lack of nationwide screening program, along with aging and growing population, probably explain the rising CRC rates.

The Kingdom of Saudi Arabia has experienced unprecedented economic and social development in recent decades, with increased per capita availability of oils and fats (200%), animal fat (171%), animal protein (207%), meat (313%), milk (120%), eggs (648%) and sugar (168%), along with increased consumption of calories and proteins by individuals, which exceeds the recommended daily allowances by 147 and 217%, respectively.[32,33] On the flip side, only 40% of Saudis eat fresh vegetables or fruits daily.[34] A national cross-sectional study showed that only 28% of adult Saudis do physical exercise three times a week. Among the Saudis, 35% are obese and 37% are overweight.[34]

Since the estimated induction/latency period for CRC may be quite long,[35] low ASRs for CRC in KSA, in the early 1990's, probably reflected the low level of exposure to environmental risk factors in the preceding two or three decades. Thereafter, rapid and progressive rising rates reflect the fast acquisition of western lifestyle, which may be associated with the financial spike all over the country. Ranked fifth among the incidences of cancer in KSA in 1994, CRC became the second, after breast cancer, in 2003. In Saudis as well as non-Saudis, CRC is the first and third cancer in males and females, respectively.

In USA, the decline in CRC mortality may be associated with the declining incidence, screening and improving disease outcomes by earlier stage diagnosis and improving cancer treatment.[29,30,36] Conversely, higher CRC mortality in Saudis, especially females, might relate to increasing incidence, lack of screening with consequent advanced stage at diagnosis, accessibility to specialized centers and high prevalence of obesity, physical inactivity and diabetes.[34,37]

Our analyses predicted a significant increase (fourfold) in the burden of CRC in KSA, in the coming decades. Health authorities should adopt parallel strategies to face the expected surge in the incidence of CRC. Slow growth in the supply health care professionals, especially oncologists, oncological surgeons, radiation oncologists and nurses is a worldwide problem.[38–41] Nurse practitioners and physician assistants may help in bringing down this shortage. Family physicians may take more responsibility in screening and early detection programs; they should be more proactive in following-up cancer patients after the patients are transferred back to their care from the oncology facilities.[42] Preventing CRC should be a goal, as many of its risk factors are related to lifestyle.[43]

There is an urgent need to make the population aware of the possible relation between diet and CRC. The challenge is to work out how best to formulate national food policies that capitalize on the usual benefits of an improved food supply and nutritional status and yet minimize the social and economic costs of diet-related chronic diseases.[32]

Screening is important in treating precancerous lesions, prior to evolution to a frank malignancy. Also, it detects the disease at an early, probably asymptomatic stage, with better outcomes. To be cost-effective, subjects are to be classified into average-, high- and very high-risk individuals.[44] Subjects at very high risk, belonging to families with hereditary transmission as familial adenomatous polyposis (FAP) and hereditary non-polyposis CRC (HNPCC), should be screened at the age of 25 or five years before the age of the earliest diagnosis in the family, as defined by the Amsterdam's criteria.[45] With one affected first degree relative before the age of 45 years or with two affected first degree relatives, screening is probably advised. Patients with prior history of CRC or large adenomas are to be screened dating from the diagnosis. Patients with ulcerative colitis or Crohn's disease should be screened, beginning 15-20 years after the development of pancolitis. For the rest of the eligible Saudi population (age 50 years or more), the value of screening is to be defined, taking into consideration the current low ASRs, albeit with a noticeable rise.

We believe that the present work should provide an impetus to study other prevalent types of cancer, particularly in the developing countries.
